# A Digital Tool (Technology-Assisted Problem Management Plus) for Lay Health Workers to Address Common Mental Health Disorders: Co-production and Usability Study in Pakistan

**DOI:** 10.2196/59414

**Published:** 2025-01-28

**Authors:** Maham Saleem, Shamsa Zafar, Thomas Klein, Markus Koesters, Adnan Bashir, Daniela C Fuhr, Siham Sikander, Hajo Zeeb

**Affiliations:** 1 Leibniz Institute for Prevention Research and Epidemiology – BIPS Bremen Germany; 2 Leibniz Science Campus Digital Public Health Bremen Germany; 3 Fazaia Medical College Air University Islamabad Pakistan; 4 Department of Psychiatry and Psychotherapy II District Hospital Guenzburg Ulm University Ulm Germany; 5 Center for Evidence-Based Healthcare Medical Faculty Carl Gustav Carus Technische Universität Dresden Dresden Germany; 6 Health Information Systems Program Islamabad Pakistan; 7 Department of Primary Care and Mental Health University of Liverpool Liverpool United Kingdom

**Keywords:** digital mental health, digital technology, digital intervention, Problem Management Plus, lay health worker programme, common mental health disorders, low- and middle-income countries, co-production

## Abstract

**Background:**

Mental health remains among the top 10 leading causes of disease burden globally, and there is a significant treatment gap due to limited resources, stigma, limited accessibility, and low perceived need for treatment. Problem Management Plus, a World Health Organization–endorsed brief psychological intervention for mental health disorders, has been shown to be effective and cost-effective in various countries globally but faces implementation challenges, such as quality control in training, supervision, and delivery. While digital technologies to foster mental health care have the potential to close treatment gaps and address the issues of quality control, their development requires context-specific, interdisciplinary, and participatory approaches to enhance impact and acceptance.

**Objective:**

We aimed to co-produce Technology-Assisted Problem Management Plus (TA-PM+) for "lady health workers" (LHWs; this is the terminology used by the Lady Health Worker Programme for lay health workers) to efficiently deliver sessions to women with symptoms of common mental health disorders within the community settings of Pakistan and conducted usability testing in community settings.

**Methods:**

A 3-stage framework was used for co-producing and prototyping the intervention. Stage 1 (evidence review and stakeholder consultation) included 3 focus group discussions with 32 LHWs and 7 in-depth interviews with key stakeholders working in the health system or at the health policy level. Thematic analyses using the Capability, Opportunity, and Motivation for Behavioral Change (COM-B) model were conducted. Stage 2 included over eight online workshops, and a multidisciplinary intervention development group co-produced TA-PM+. Stage 3 (prototyping) involved 2 usability testing rounds. In round 1 conducted in laboratory settings, 6 LHWs participated in role plays and completed the 15-item mHealth Usability App Questionnaire (MUAQ) (score range 0-7). In round 2 conducted in community settings, trained LHWs delivered the intervention to 6 participants screened for depression and anxiety. Data were collected using the MUAQ completed by LHWs and the Patient Satisfaction Questionnaire (PSQ) (score range 0-46) completed by participants.

**Results:**

Qualitative analysis indicated that a lack of digital skills among LHWs, high workload, resource scarcity for digitization (specifically internet bandwidth in the community), and need for comprehensive training were barriers for TA-PM+ implementation in the community through LHWs. Training, professional support, user guidance, an easy and automated interface, offline functionalities, incentives, and strong credibility among communities were perceived to enhance the capability, opportunity, and motivation of LHWs to implement TA-PM+. TA-PM+ was co-produced with features like an automated interface, a personal dashboard, guidance videos, and a connected supervisory panel. The mean MUAQ score was 5.62 in round 1 of usability testing and improved to 5.96 after incorporating LHW feedback in round 2. The mean PSQ score for TA-PM+ was 40 in round 2.

**Conclusions:**

Co-production of TA-PM+ for LHWs balanced context and evidence. The 3-stage iterative development approach resulted in high usability and acceptability of TA-PM+ for LHWs and participants.

## Introduction

### Background

Recent research has indicated that mental health remains among the top 10 leading causes of disease burden globally and constitutes 32.4% of years lived with disability [1]. Common mental health disorders are conditions that impact mood, thought processes, and behavior, often leading to challenges in daily functioning and overall well-being. These include disorders, such as depression, anxiety, and bipolar disorder, each characterized by unique symptoms and impacts on the lives of individuals [2]. Depression, affecting over 300 million people globally, is the most prevalent mental health disorder [3]. A major part of this burden (about 70%) is concentrated in low- and middle-income countries (LMICs). Additionally, a shortage of mental health specialists in LMICs contributes to a mental health treatment gap of 90% for almost all mental health conditions, meaning that only 1 out of 10 conditions requiring mental health treatment receive it from a mental health specialist [3]. Various factors contribute to the mental health treatment gap, including lack of human resources for mental health, stigma, accessibility, uneven mental health service distribution, and lack of a perceived need for treatment [4]. To address this gap, the World Health Organization (WHO) developed the Mental Health Gap Action Programme (mhGAP) by providing evidence-based interventions for 8 priority mental health disorders. These guidelines recommend involving lay health workers and community members in the early detection of mental health disorders and low-intensity psychological care of patients with mental health disorders. This can help LMICs address the treatment gap and health resource shortfall, and achieve WHO Mental Health Action Plan targets [3,4]. Building on the WHO’s mhGAP, several initiatives in LMICs have been developed to address the mental health treatment gap [5-7]. These programs empower nonspecialist health workers to deliver mental health care through structured culturally adapted interventions. Examples include Problem Management Plus (PM+), a transdiagnostic intervention for managing stress and depression, and the Thinking Healthy Programme, which addresses perinatal depression through simplified behavioral strategies. Community-based programs like Friendship Bench in Zimbabwe further extend the reach of mhGAP by embedding mental health support within local settings, demonstrating significant improvements in access and patient outcomes. The Disease Control Priorities (DCP3) report endorses such scalable models as effective affordable solutions that bolster mental health care capacity in LMICs [6,7]. A comprehensive review [7] of 208 studies focusing on various mhGAP interventions in LMICs demonstrated their overall efficacy. The mhGAP interventions were shown to significantly improve the symptoms of common mental health disorders, reduce substance use, and positively impact parenting and child outcomes. Furthermore, mhGAP training across these interventions enhanced the clinical skills, knowledge, and competency of health workers, contributing to improved quality of care after training [7].

PM+ is a low-intensity brief psychological and transdiagnostic intervention for common mental health disorders. It is an evidence-based intervention that can address various mental health issues and address the limitations posed by scarce specialized treatments in low-resource settings [8-14]. PM+ has been designed for depression, anxiety, stress, grief, and self-identified practical issues (interpersonal conflict and unemployment) and is delivered by lay health workers [15]. The intervention consists of 5 weekly face-to-face sessions involving stress management and problem-solving approaches [15]. PM+ has been tested for effectiveness and cost-effectiveness in various LMICs [8-14]. It has proven to be particularly effective in reducing the symptoms of anxiety, depression, and general distress in settings affected by significant adversity, including conflict-affected regions or under-resourced settings [16]. PM+ is recommended for stepped-care models, especially in low-resource settings where it serves as an effective first-line intervention. In these contexts, PM+ addresses common mental health needs at a low cost, allowing higher-intensity services to be reserved for more severe cases. This approach optimizes resource use and enhances accessibility in areas with limited mental health infrastructure. Notably, PM+ is available in several languages including Urdu, and it has been contextualized and rigorously evaluated in Pakistan [10,12,13].

However, the implementation of PM+ can be challenging for lay health workers and can be hampered by issues of quality control in training, supervision, and delivery [8-11]. Lengthy sessions and the complex nature of systematic stress and patient problem management strategies further compound these challenges, along with the struggle to recall all core strategies and protocols [8-11]. This specific phenomenon is termed “program drift” in the field of implementation science, which refers to a situation where the quality and fidelity of an intervention diminish because of deviations from the manual protocol, resulting in a reduction in the potential benefits of the intervention [17]. These challenges and barriers can be overcome by using digital tools and training lay health workers for them [18]. A recent systematic review of studies from LMICs revealed that the integration of digital technologies into mental health interventions led by nonspecialists typically results in positive outcomes and has the potential to address the following three gaps highlighted in mental health literature: (1) the treatment gap, (2) the treatment-quality gap by improving the competencies and knowledge of nonspecialists, and (3) the service utilization gap by increasing the use of mental health care services [18].

Few initiatives have pursued digital adaptations of PM+ to enhance accessibility and scalability, particularly in low-resource settings. The step-by-step intervention was initially conceptualized as an online self-help adaptation of PM+. While the step-by-step intervention initially incorporated the principles of PM+, the intervention diverged as the complexity of implementing problem-solving components within a digital app proved challenging. Consequently, the step-by-step intervention evolved into an independent transdiagnostic intervention focused on managing stress and addressing depression and anxiety through behavioral activation principles [19]. In response to the COVID-19 pandemic, additional digital training frameworks were developed to enable remote training of lay health workers, ensuring uninterrupted mental health support amid physical distancing measures [20].

PM+ has been rigorously evaluated in Pakistan, showing effectiveness in reducing depression and anxiety symptoms [10,12,13]. Despite higher initial costs than the standard care, it has demonstrated long-term cost-effectiveness and is recommended for integration into a stepped-care model in resource-limited settings like Pakistan [12]. In a stepped-care model [21], individuals begin with lower-intensity support and progress to more intensive treatments only if necessary, optimizing resources and tailoring care to individual needs. This approach is particularly relevant in Pakistan, where mental health services are limited and mostly concentrated in major urban centers [22]. Pakistan’s health care system, with its community outreach through "lady health workers" (LHWs; this is the terminology used by the Lady Health Worker Programme for lay health workers) [23], who have established supervision and referral mechanisms, provides a strong foundation for implementing stepped-care mental health interventions like PM+. To address the challenges in implementing PM+ by applying technological assistance and to align with the recommended stepped-care model for Pakistan, we aimed to develop a technological platform Technology-Assisted Problem Management Plus (TA-PM+). TA-PM+ consists of an app designed to support nonspecialists in delivering the PM+ intervention within Pakistan’s health system, with an established backend for supervision, monitoring, and referral processes. TA-PM+ seeks to ensure the consistent delivery of therapeutic components at the appropriate dosage and with high fidelity while being delivered face-to-face, fostering a therapeutic alliance, which is essential for maintaining the problem-management focus required for effective intervention delivery. This paper describes the stepwise co-production of TA-PM+ and its key features.

### Objective

We aimed to co-produce TA-PM+ for LHWs to use in efficiently delivering sessions to women within community settings in Pakistan.

## Methods

### Setting and Context

The study was conducted in the Federal District of Islamabad Capital Territory (ICT), Pakistan. The study considered the Union Council (UC) of Tarlai, which has a population of 100,000 and is located about 10 kilometers outside the ICT. The UC is a predominantly low-income and lower middle–class area, with a significant number of migrants from different parts of Pakistan. The crude birth rate is estimated to be around 40 births per 1000 population, with families typically having 4-8 children. Traditional gender roles prevail in families. Among the poorer sections of the population, several adversities are common, including malnutrition, irregular income, and low literacy rates [24].

Each UC is served by the primary health care unit, which consists of a medical doctor, vaccinators, and a pharmacist, and serves as the base for the community outreach program of LHWs. The LHWs are supervised and monitored by a facility-based supervisor, known as the lady health supervisor (LHS). The LHWs provide family planning, and maternal and child preventive and promotive services to 250-300 households in the catchment area and establish referrals for high-risk cases. More recently, maternal and adolescent mental health awareness and prevention have been added to the curriculum. The UC of Tarlai has 32 LHWs who cover nearly 80% of the population of Tarlai.

### Ethical Considerations

This study has been approved by the Institutional Review Board of Fazaia Medical College, Islamabad, Pakistan (approval number: IBD/FMC/1341/IRB), in accordance with the ethical guidelines for human subject research and the Declaration of Helsinki. Informed consent was obtained from all participants, and for secondary analyses, the original consent permitted data use without requiring additional permissions. All data were anonymized to ensure privacy and were securely stored on a protected cloud platform accessible only to the research team. Participants received modest compensation, such as financial allowances and travel reimbursements (US $5-7). No identifiable participant information has been included in the manuscript or supplementary materials.

### TA-PM+ Co-production

In line with the 3-stage framework of co-production and prototyping of public health interventions [25], TA-PM+ was designed in three stages: (1) qualitative investigation, (2) co-production, and (3) prototyping and usability testing. Qualitative investigation used semistructured interviews and was designed and reported in line with the Consolidated Criteria for Reporting Qualitative Research (COREQ) checklist (Multimedia Appendix 1).

#### Stage 1: Qualitative Investigation

A qualitative study was conducted to inform the development of TA-PM+. The main objectives were to explore implementation barriers; understand user needs (including training and supervision), particularly those related to technology use assisting intervention delivery; evaluate monitoring; and identify TA-PM+ functionalities that could be used to strategically address barriers and aspects of delivering PM+ sessions. The sample for qualitative investigation consisted of LHWs, an LHS, and participants working in the health system or at the health policy level. Focus group discussions and in-depth interviews were conducted as part of qualitative investigation. All participants were given information about the research aims, and rapport was established.

##### Focus Group Discussions

A trained female researcher conducted 3 focus groups between June and August 2022 with the LHWs (n=32). They were purposefully sampled from a range of settings to ensure diversity in demographic factors, age, and education. The focus group discussions were held at a primary health care facility in Islamabad, Pakistan. One focus group discussion lasted between 70 and 85 minutes. The focus group discussions were held in Urdu, and data on mental health, digitization, and intervention functions perceived to be useful for TA-PM+ were elicited using semistructured techniques (Multimedia Appendix 2).

##### In-Depth Interviews

Interviews were conducted with a purposive sample of 7 participants working in the health system (n=2) or at the health policy level (n=5) between July and August 2022. The participants included an LHS, doctors at the primary health care unit of the health system, health system specialists, individuals in Director General programs, program officers of noncommunicable diseases, and digital health and information specialists at the policy level. The interviews were conducted in Urdu and face-to-face at a location of the participant’s choice by a trained female researcher. The duration of the interviews ranged from 35 to 45 minutes, during which a semistructured interview guide was employed (Multimedia Appendix 2).

##### Data Analysis

The focus group discussions and interviews were audio recorded and transcribed verbatim. The framework analysis approach was used for analyses consisting of familiarization, identification of the thematic framework, charting, mapping, and interpretation. A second author (SZ) was engaged to independently code a randomly selected subset of 20% of the transcripts. The deductive framework was constructed using the Capability Opportunity and Motivation for Behavioral Change framework and the Theoretical Domain Framework (TDF) [26]. The TDF was implemented in deductive analyses to include various elements that otherwise might have been ignored. However, the researcher realized that not all constructs would be used to code the data, and constructs that did not yield any findings were excluded. The thematic framework was refined through repetitive discussions between the researchers MS and SZ. To ensure the authenticity of the data and to minimize the risk of data loss, the coding process was carried out on the transcript written in Urdu. Once indexing and charting were completed using MaxQDA, the coded sections of the transcript were double translated into English.

#### Stage 2: Co-production of TA-PM+

During stage 2, TA-PM+ was collaboratively developed by an intervention development group comprising both local (n=5) and international (n=5) members. This group included professionals with diverse backgrounds, including software developers, clinical psychiatrists, epidemiologists, public health researchers, and health system specialists.

##### Co-production Workshops

Eight co-production workshops were conducted online on Zoom between July and October 2022. Each co-production workshop lasted 120 to 150 minutes and was conducted with the aim of planning TA-PM+ features and delivery mechanisms. The findings from stage 1 were presented, ideas were sought from all members, and a storyboard was formulated. Subsequently, software designers presented concepts, prototypes, and avatar samples, and this was followed by the collection of feedback and iterative refinement, ultimately resulting in the finalization of features for TA-PM+. All the workshops were recorded, and after each workshop, a researcher (MS) summarized the findings decided during the workshop, which included technological features, scripts of the videos from the PM+ manual, and text for different interfaces of sessions. Additionally, member checking [27] (ie, sharing the findings with all members of the workshop) was carried out to verify the authenticity of the summarized findings. The finalized summary was shared with the software designers who prepared diagrams, vignettes, icon pictures, and interface mock-ups and was presented at the next workshop where feedback was sought again. This process was replicated across all 8 workshops, resulting in the finalization of all features, interface mock-ups, and videos for TA-PM+.

##### TA-PM+ Technological Platform Development

TA-PM+ was developed using the Agile methodology fostering flexibility and adaptability throughout the app’s development and maintenance phases [28] from October 2022 till May 2023. The Agile methodology is an iterative approach to software development that prioritizes flexibility, customer collaboration, and continuous improvement. It emphasizes adaptive planning, incremental delivery, and embracing change. Agile teams work in short iterations to deliver working software increments, gather feedback, and adapt to evolving requirements. TA-PM+ is designed to operate on standard mobile devices, including iOS and Android platforms, underpinned by a robust hardware infrastructure. The mobile app and web dashboard are hosted on servers that necessitate a configuration with a minimum of 4 GB of RAM and 20 GB of storage capacity, ensuring the smooth operation of the app. MySQL is employed as the database management system on the server, providing a reliable platform for data management.

The software components of TA-PM+ are developed using advanced programming languages. PHP 7.3 and PHP 8.0 are leveraged for web development, while React Native is used for the mobile app and Laravel 8.40 is used for web development.

The TA-PM+ mobile app, developed using React Native, serves as the primary interface for users. The server-side component, developed using PHP (Laravel), involves a RESTful API and is responsible for authenticating users, conducting assessments, and facilitating data retrieval. The web dashboard, built using PHP (Laravel), is accessible via web browsers and is primarily intended for administrators. It provides a user-friendly interface for content management, configuration settings, and report viewing.

#### Stage 3: Prototyping and Usability Testing

##### Prototyping

After the co-production, external experts (n=2) in the PM+ intervention and in clinical psychiatry prototyped the content, resources, and videos of TA-PM+. The content was updated according to their feedback. The intervention development team examined the application for heuristic evaluation employing Jakob Nielsen heuristics [29].

##### Cultural Adaptation

We used the culturally adapted and Urdu-translated PM+ manual developed for the Pakistan context [10,12,13]. To further enhance cultural relevance, psychoeducational videos for patients were developed with detailed adjustments, including culturally appropriate avatars, dress codes, names, video backgrounds, and case scenarios. Voiceovers were provided by a native Urdu speaker, and the videos were reviewed by clinical psychiatrists (n=2) in Pakistan for language refinement. LHWs (n=6) participated in usability testing of TA-PM+, providing feedback on wording, icon labels, and flowcharts in the app. Additionally, women (n=6) from the community together with LHWs (n=6) evaluated the videos for cultural appropriateness and sensitivity. All feedback was incorporated to optimize clarity, usability, and cultural alignment.

##### Usability Testing

To evaluate the preliminary acceptance and utility of TA-PM+, usability testing sessions were carried out with a convenience sample of LHWs (n=6) in 2 rounds. Nelson [30] has established that a total of 5 participants is sufficient to detect usability issues.

##### Recruitment

Six LHWs were randomly recruited from the primary health care unit at Tarlai for usability testing. The LHWs had a comprehensive training program of 15 hours, which focused on their proficiency in using TA-PM+. The training agenda included introduction to common mental health disorders, confidentiality, PSYCHLOPS (Psychological Outcome Profiles), introduction to the PM+ intervention, content of the first session of PM+ on stress management, and delivery of the first session using TA-PM+. Subsequently, for round 2 of usability testing, recruitment was conducted in community settings, and the LHWs identified individuals exhibiting symptoms indicative of a common mental health problem from their catchment areas. A researcher screened these women for the symptoms of common mental health disorders. The following two screening tools were used (specific cutoffs were applied): (1) General Health Questionnaire-12 (GHQ-12): a score of 3 or higher on the GHQ-12, which is a 12-item questionnaire measuring general psychological distress on a 4-point scale (possible range of 0 to 36) and (2) WHO Disability Assessment Schedule (WHODAS): a score of 16 or higher on the WHODAS 2.0, which is a screener for functional impairment consisting of 12 items measured on a scale ranging from 1 to 5 (possible range of 12 to 60).

A total of 6 women from community settings were included for round 2 of usability testing.

##### Data Collection

The LHWs completed round 1 of usability testing through role plays following the completion of the training, using the mHealth App Usability Questionnaire (MUAQ) for standalone apps for health care providers [31]. The MUAQ comprises 18 items rated on a 7-point scale, with a higher score indicating greater usability. To assess the usability of TA-PM+, the total average score of the responses to all statements was calculated. With a maximum score of 7, a higher overall average score indicates superior app usability. Feedback was sought from LHWs, and TA-PM+ was improved with feedback gathered before round 2 of usability testing was conducted in the community with 6 participants. After recruiting participants, 6 trained LHWs delivered the first session of TA-PM+ to the positively screened individuals. After session delivery, the MUAQ [31] was administered to the LHWs, while the Patient Satisfaction Questionnaire (PSQ) involving an mHealth intervention [32] adapted for TA-PM+ was administered to the participants. The PSQ comprises a total of 14 items. On a 5-point Likert scale, respondents indicate the degree of agreement with each item. Ten items are presented in the positive, while 4 are stated in the negative. In the analysis, the negatively stated items are inverted; therefore, a higher score indicates increased satisfaction with the intervention. The highest possible score is 46. Another round of feedback was gathered from the LHWs and integrated into the final version of the TA-PM+ app.

## Results

### Overview

This section is divided into three parts describing the results of (1) qualitative investigation, (2) co-production, and (3) prototyping and evaluation.

### Qualitative Investigation

#### Participant Characteristics

A total of 40 participants (mean age 49 years; range 40-60 years) were recruited, out of which 34 were female and 6 were male. Among the 40 participants, 30 completed 10 years of education, 2 completed 14 years of education, and 8 completed over 16 years of education.

#### Perception of Factors Influencing the Implementation of TA-PM+ and the Features of TA-PM+ to Address These Factors Within the Capability, Opportunity, and Motivation-Behavior Model

**Figure 1:**
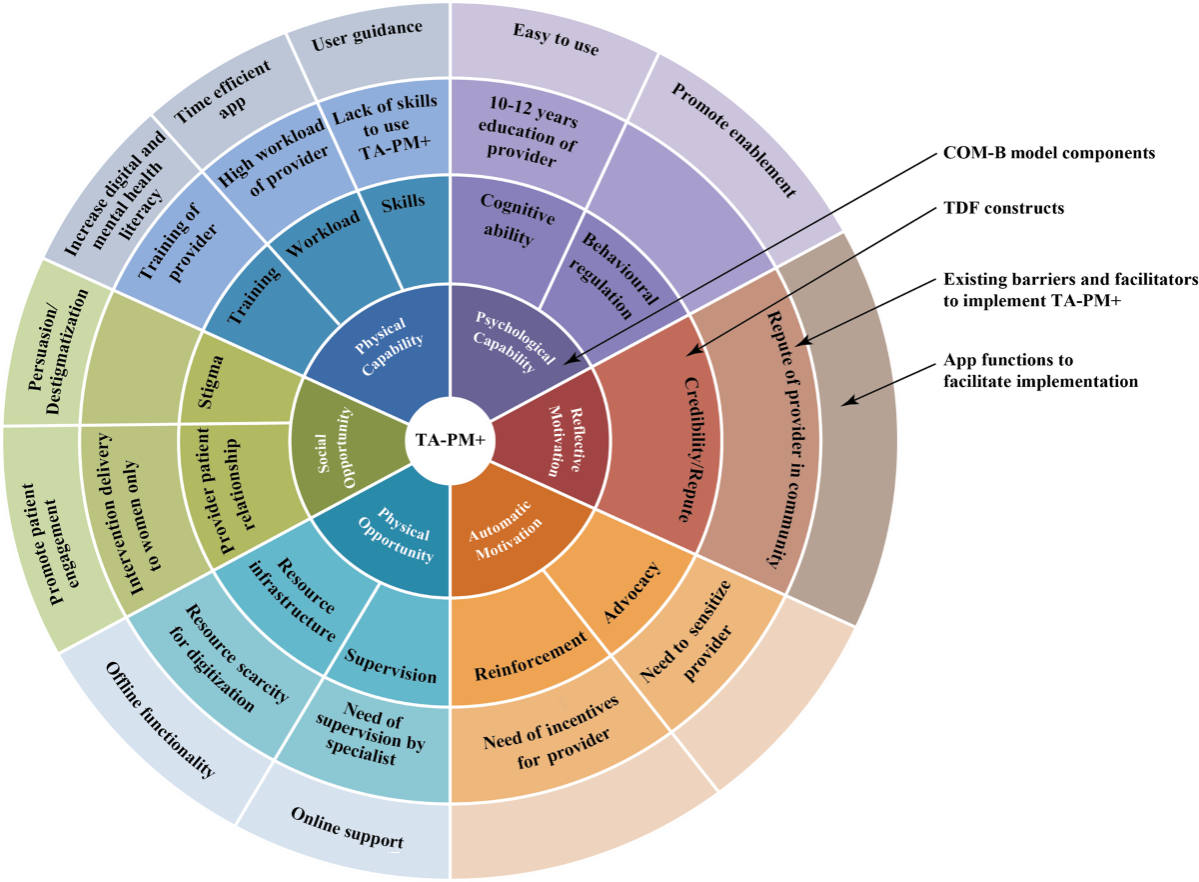
Perceived barriers and facilitators for the implementation of Technology-Assisted Problem Management Plus (TA-PM+) and the corresponding digital facilitators of TA-PM+ identified to address the barriers. Thematic analysis conducted using the Capability, Opportunity, and Motivation for Behavioral Change (COM-B) framework. TDF: Theoretical Domain Framework.

Figure 1 provides an overview of the health system’s barriers to the implementation of TA-PM+ and the TA-PM+ features to facilitate implementation that were mapped under the constructs of the TDF and the components of the Capability, Opportunity, and Motivation for Behavioral Change (COM-B) model. The code chart with the description of codes is provided in Multimedia Appendix 3.

##### Barriers and Facilitators of the Health System Capacity to Implement TA-PM+ and the Features of TA-PM+ to Improve the Capacity Within the COM-B Framework

###### Knowledge and Skills

The significance of the knowledge and skill proficiency of LHWs in the effective implementation of TA-PM+ was highlighted by multiple participants. Although the digitization of the outpatient departments (OPDs) of LHWs has resulted in the development of some competencies to use digital tools, this aspect assumes utmost importance in the implementation process. Quotes have been provided, and the position of each quote in the transcript has been indicated.

There are some challenges for this intervention. Although the LHWs are given tablets, and their OPD is digitized…they receive proper digital training. But then again there might be problems regarding digital skills of LHWs to implement TA-PM+.

Health policy participant_5, Position 10

The challenges that can be faced include HR (Human Resources) and capacity issues.

Health policy participant_3, Position 50

Many participants perceived that in addition to training, the provision of user guidance and the incorporation of a fully automated guided app could effectively address the competency barrier.

When we open this app, it guides us from beginning till end. For example, when we are checking a child in a family, we open it all data comes and as we keep progressing, we are instructed what to do like its written ‘check' and the name of the medicine, we click it, and it moves forward and tells us what to do next. This will make it easier for us.

Focus group_1, LHW participant_6, Position 110

###### Time/Workload of the Provider

All the participants agreed that a significant barrier to the implementation of TA-PM+ is the workload of LHWs. They were extensively involved in health promotion, disease prevention, and care delivery to women and children in their catchment area. The integration of a complex psychological intervention was perceived as an additional burden. Few respondents believed that the added workload associated with the additional digital tool complexity would outweigh the assistance TA-PM+ is expected to provide in delivering the intervention.

We visit seven to eight homes daily, work on various aspects. We spend around 40 to 50 minutes in one household. In our app, which is dedicated to dengue, we send pictures. We also monitor the weight of children and assess the health of both mothers and children. When we encounter pregnant women, we provide them with cards and refer them accordingly. We give guidance, suggesting that they go to the center and obtain medication from there. In addition to all of this, we also record everything in our registers, and we enter data in the tablets.

Focus group_3, LHW participant_1, Position 3

The proposed design and strategies aimed at mitigating the strain experienced by LHWs were primarily focused on improving the time efficiency of TA-PM+. The participants strongly agreed on the importance of minimizing the amount of time spent using TA-PM+. Moreover, the participants emphasized the need for a simplified method that alleviates LHWs from the burden of typing. The ideal app, according to the participants, would be one that is fully automated and requires minimal input from LHWs. Almost all participants agreed to reduce session durations by leveraging the capabilities of the digital platform.

First, it should let them do everything with just a click. Why not create a form in such a way that these poor women don't need to type? So, I would suggest that you consider developing an app that is quick for them to use. The reason behind this suggestion is that these women are dealing with dengue, pneumonia, polio, health education, breast feeding, breast cancer, family planning, and post-abortion matters. So, think about how it could benefit them.

Health system participant_6, Position 100

###### Training

The importance of LHW training was acknowledged by the participants as a critical component of successful implementation. They emphasized the significance of comprehensive training covering the PM+ intervention as well as training to use TA-PM+. Some participants also highlighted the importance of field usability testing prior to implementation.

They should be guided on how to use it properly, and proper training and facilitation will be required. Train them for problem management plus and for the digital App. I think they shouldn't encounter any problems, but essentially, it's about sensitizing them first, then explaining the application thoroughly and conducting proper training. And, useful to conduct field test, then it should work well.

Health policy participant_5, Position 16

Regarding TA-PM+ functionalities, the participants felt that it should improve the TA-PM+ digital literacy of LHWs. Suggestions included the use of infographics or the provision of TA-PM+ introduction videos to serve as a refresher training for LHWs. This functionality was thought to be a helpful tool that LHWs could access at any time to address challenges in using the app.

Infographics should be designed in such a way that the LHWs can easily understand and get refresher training. For example, the LHW goes into the community, and sometimes someone asks her how to make ORS (Oral Rehydration Solution), and she doesn't remember how to make ORS. So, if she opens her learning platform and she can see how to make it. If there's a small infographic video uploaded, it will make it easier for community workers. Sometimes they may not remember everything, but with such resources, they can easily learn.

Health policy participant_3, Position 56

###### Cognitive Capacity

The cognitive capacity, including memory and attention, of LHWs has been identified as a crucial factor in the successful implementation of the TA-PM+ intervention. The majority of LHWs have 10 years of formal education. After training, LHWs need to remember and retain the information provided, pay attention to the needs and concerns of the individuals receiving the intervention, and actively engage with TA-PM+ to effectively deliver the intended support. Given the complexity of TA-PM+ and the cognitive demands associated with delivering it through a digital platform, it is crucial to develop an easy-to-use app that is not overly complex and employs plain language.

These poor women are not IT experts, they are all over 40, and while it seems easy, at this age, learning something new is challenging for them because they are accustomed to their old ways. So, I would suggest that you provide them with an app that is easy to download and use.

Health system participant_6, Position 92

###### Behavioral Regulation

One of the most important functionalities of TA-PM+ highlighted by LHWs was to promote enablement, which refers to the app’s capacity to enhance precision in intervention delivery. To ensure that the intervention is implemented successfully, the TA-PM+ design must be optimized to promote and support the targeted actions.

At first, the name should appear, then their age, followed by their phone number, and then some other contact information. Also, session information like you've started a session, an entry has been made for that session or our visit data is recorded in it. The next session and date should be visible and whenever we click on patient name, the next session will come up.

Focus group_3, LHW participant_6, Position 145

##### Barriers and Facilitators for the Opportunity in Health Systems to Implement TA-PM+ and the Features of TA-PM+ to Increase the Opportunity Within the COM-B Framework

###### Resources/Infrastructure

The effective implementation of TA-PM+ relies on the availability of sufficient infrastructure and resources. The ongoing health reforms and digitization of health care in Pakistan provide a favorable environment for the implementation of a digital mental health intervention. However, the digitization of primary health systems is still in its early stages, and LHWs face numerous challenges. Despite the availability of digital tablets and the use of digital applications by LHWs for service delivery, concerns remain regarding device insurance, internet availability in different areas, and limited funds for internet access.

When you go to the community, sometimes it happens that you don't have access to the internet, and we cannot submit data. This internet, which is making life easy for people, has become a burden for us. It has become a source of trouble.

Focus group_1, LHW participant_8, Position 254

One of the key features considered important by all participants was offline functionality for TA-PM+. It was unanimously agreed that TA-PM+ should not rely on internet connectivity for its operation.

###### Monitoring, Supervision, and Support

The provision of supervision and continuous monitoring of LHWs was seen as crucial to the effective delivery of TA-PM+ to mental health patients. Furthermore, it is of utmost importance to ensure adequate support in case of any issues arising with the app.

The current model, which involves the supervision of LHWs by LHSs and referral from the community to primary health care facilities as needed, has been identified as a favorable approach for implementation. For TA-PM+ functionality, the online monitoring and referral features should be included to enhance the supervision process and ensure efficient communication between LHWs and health care facilities.

There should be a referral system in place where there is someone available who can assess if your patient is not benefiting from sessions and if their condition is severe, they should be referred through app. There should be a doctor available at the center, and there should be a clear referral process. It should also be made convenient so that if someone comes for help, it is beneficial for them, and there is no embarrassment for us.

Focus group_3, LHW participant_2, Position 276

##### Patient and Provider Relationship

TA-PM+ will be delivered by LHWs, and all participants perceived that the delivery can only be planned for women residing in the areas. Delivering the intervention to men is not possible given the focus of the LHW program on women’s health. Furthermore, it was also perceived as a facilitator to implementation because LHWs have a positive reputation among the women of the community and the entire household has trust in them. This will facilitate the identification of cases of depression and stress by the LHW, as she will be fully informed of the circumstances a woman is experiencing.

When you visit someone's home, you get an idea of the internal situation there. You can estimate how they are doing, for instance... by observing their tone, silence, arguments, and fights. You can figure out that if a woman is distressed now, it might be because of her husband, or maybe because of her mother-in-law. In this case, they can obtain history and they can provide this lady intervention. And I think that it's not easy to go to a psychologist or psychiatrist for stress. But lady health worker can deal with this individual very comfortably.

Health system participant_6, Position 108

Participants indicated that features that improve patient engagement with the intervention were needed in TA-PM+ functionality. Features that are specifically tailored for patients and actively encourage and support patient engagement should be included in TA-PM+ to enhance the effectiveness of the intervention and further strengthen the patient-provider relationship.

We have worked on another digital project, there were videos for patients in that. We used to show them videos initially, and patient would understand quite well and used to be very convinced and focused.

Focus group_3, LHW participant_2, Position 130

###### Stigma

Stigma surrounding mental health disorders persists within the society in Pakistan, leading many individuals to perceive mental health treatment as stigmatized. Mental health services are generally provided at tertiary health hospitals, and psychiatric wards are locally known by stigmatized labels, reinforcing mental health’s negative connotations. However, an alternative approach, that is, TA-PM+ provision in the community, specifically in the patient’s home with LHWs, may reduce stigma and encourage treatment. This is because the community trusts these LHWs. One important component of TA-PM+ that has been identified is the use of persuasive strategies to reduce mental health stigma. In addition to promoting user engagement, TA-PM+ should also destigmatize mental health disorders and convince patients to accept support.

I really liked the feature of videos, especially for LHWs dealing with depression patients. This is very good. Additionally, there should be a face-to-face component in it. But overall, if you create something that includes engaging and persuading videos for patients, I think it would be very easily available to them all the time through the digital platform. I think it's good enough and efficient enough.

Health policy participant_7, Position 11-12

##### Barriers and Facilitators of Motivation for Health Systems to Implement TA-PM+ and the Features of TA-PM+ to Enhance Motivation Within the COM-B Framework

###### Credibility

Critical determinants of reflective motivation include credibility and reputation within the community. An LHW provides service to 150-200 households in the community where she resides. Improving her reputation in the community is directly linked to the provision of modern tools and expanded services, which leads to a sense of empowerment. It was perceived that her reputation and position in the community would be further enhanced by the implementation of the TA-PM+ program using a digital tablet to address depression and stress.

One thing to remember is that when we provide a tool to LHW, I give an example like this: if we introduce a new intervention that we are implementing throughout Pakistan, where they check blood pressure and refer patients with hypertension. When you provide them with a tool, their credibility in the community improves. In this, basically counseling, is included. But when you give them a digital tool, it motivates them and allows them to feel a bit empowered.

Health policy participant_2, Position 42

#### Co-production of TA-PM+

During the co-production of TA-PM+, the intervention development team reflected on the findings from stage 1 and decided on the key features of TA-PM+. Table 1 summarizes the TA-PM+ features planned during co-production workshops informed by the themes identified in the qualitative analyses of stage 1.

**Table 1:** Functionality of Technology-Assisted Problem Management Plus (TA-PM+) identified through a qualitative investigation and features developed in a co-production workshop.

TA-PM+^a^ functionality identified in a qualitative investigation	TA-PM+ features developed in a co-production workshop
User guidance	Various prompts and alerts in TA-PM+ to guide LHWs^b^ and confirm completion of tasks Instruction manual for TA-PM+
Easy and time efficient	Basic consistent design across every session Fully automated
Increase digital and mental literacy	Two-minute informational videos for LHWs that provide information and outline the purpose of the session, its steps, the technique used, and how to use TA-PM+ to conduct the session PDFs of the PM+^c^ Urdu manual
Reduce cognitive load	Fully automated PSYCHLOPS^d^ displays questions in accordance with the scoring protocol No typing required and audio recording available
Promote competence/enablement	Personalized dashboard for each LHW, which includes patient details, name, address, session details, and next appointment date Red, yellow, and green color codes to encourage regular appointments; the green code indicates that the session has just been delivered, the yellow code indicates that it is almost due, and the red code indicates that it is urgently due
Internet independent	To enable use in places where internet access may be costly or where bandwidth is limited or unpredictable, the content’s file size is kept as small as possible All videos are stored in TA-PM+ and can be viewed without the internet
Supervision and monitoring	Web-based supervisor backend; information is accessible regarding the session’s date, duration, PSYCHLOPS responses, and score Notification if the PSYCHLOPS score rises in subsequent sessions or suicidal ideation is noted in the patient A locking feature that prevents session data from being altered once it is submitted WhatsApp support group for urgent queries related to TA-PM+
Promote engagement	Psychoeducation videos for patients that explain the physiological symptoms of anxiety and depression to the patients
Persuasion/destigmatization	Psychoeducation videos for patients that include destigmatized vocabulary for symptoms Psychoeducation videos for patients narrated by a female doctor to boost persuasiveness

^a^TA-PM+: Technology-Assisted Problem Management Plus.

^b^LHW: lady health worker.

^c^PM+: Problem Management Plus.

^d^PSYCHLOPS: Psychological Outcome Profiles.

##### TA-PM+ User Interface

**Figure 2:**
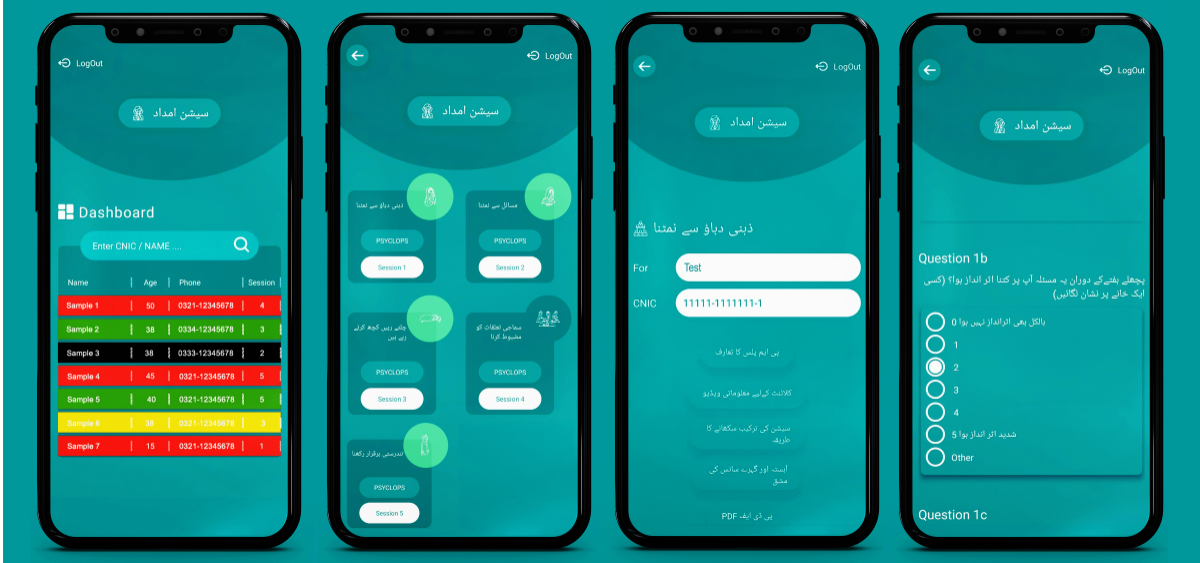
User interface of the Technology-Assisted Problem Management Plus (TA-PM+) app developed to assist lady health workers in delivering mental health interventions within community settings in Pakistan. The first screen presents a dashboard with color-coded indicators (red, yellow, and green) to track and prioritize patient sessions. Subsequent screens illustrate session navigation, patient data entry, and automated PSYCHLOPS (Psychological Outcome Profiles) assessments in Urdu. Features have been co-designed for cultural relevance and usability among lady health workers in Pakistan.

TA-PM+ is an app that guides LHWs to deliver the PM+ intervention in a systematic manner. TA-PM+ features a personalized password-protected account and personal dashboard for each LHW (Figure 2), presenting essential patient details, session specifics, and upcoming appointment dates. Using a color-coded system, the app encourages the regular scheduling of appointments, indicating the urgency of upcoming sessions. By clicking on a patient name in the dashboard, the session home screen of the patient opens. TA-PM+ ensures the security and integrity of data through its locking features, preventing the alteration of submitted data.

**Figure 3:**
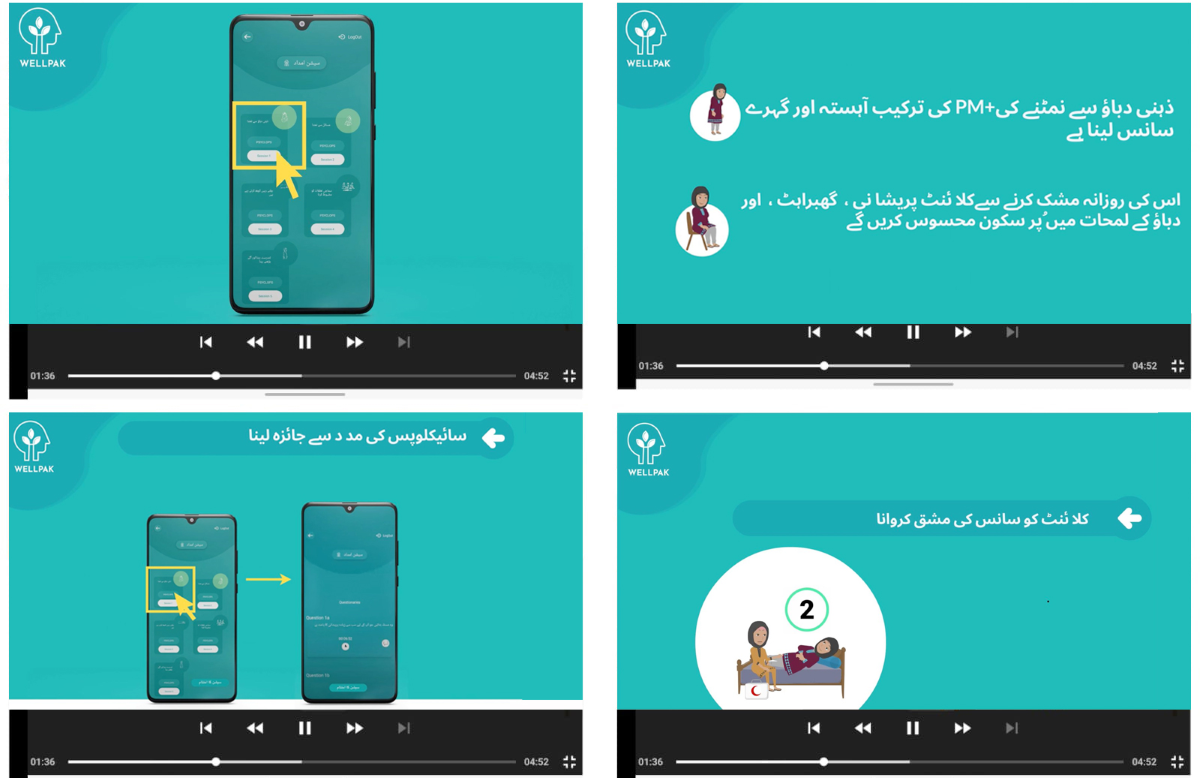
Screenshots from session-aid videos developed for lady health workers to enhance digital literacy and support the delivery of Technology-Assisted Problem Management Plus (TA-PM+). These videos, designed in Urdu, provide step-by-step instructions for navigating the TA-PM+ app and conducting sessions effectively.

TA-PM+ includes 5 informational videos, referred to as “Session Aid” (Figure 3), designed to provide LHWs with an outline of each session’s purpose, the steps involved, and the techniques employed for each session. Moreover, the videos offer guidance on how to effectively use TA-PM+ to conduct these sessions. The videos have a duration of 2 minutes to promote utilization anytime uncertainty arises, without concern for schedule delays. Additionally, TA-PM+ provides access to the PM+ Urdu manual in PDF format.

**Figure 4:**
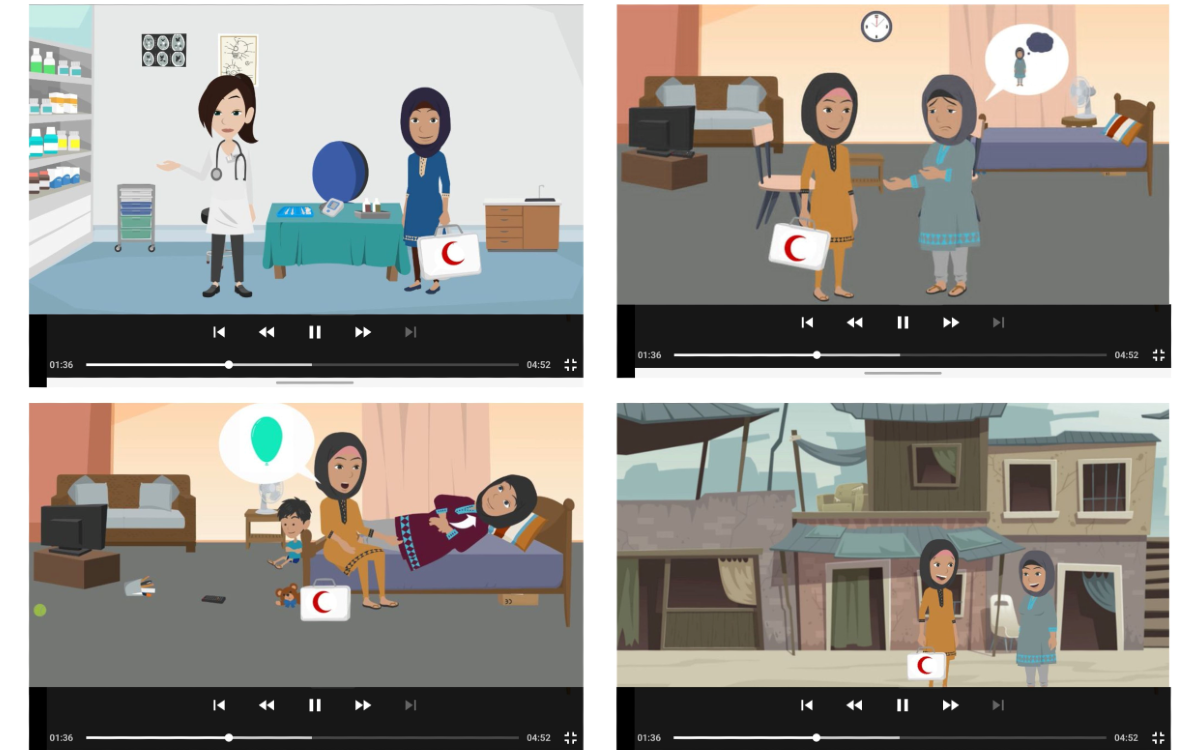
Screenshots from psychoeducational videos for patients, showing the characters Dr Ayesha, lady health worker Shazia, and patient Saima, which have been developed for sessions of Technology-Assisted Problem Management Plus (TA-PM+).

To enhance understanding and engagement, and address mental health stigma, a series of psychoeducational videos (Figure 4) were developed and included in the session interface. These videos are narrated by a fictional character Dr Ayesha and employ a narrative approach, presenting the physiological symptoms of anxiety and depression in layperson’s terms. The content is tailored to respect the cultural nuances and demographics of target communities and thus ensures maximum relevance. Using destigmatized vocabulary, the videos serve as a potent educational tool, addressing patient concerns and mitigating potential embarrassment or fear associated with their conditions. The primary objective is to develop trust and empathy, thereby promoting patient engagement and fostering acceptance of the sessions. Additionally, these videos play a vital role in efficiently explaining the active ingredients of PM+ sessions to the patients, empowering LHWs to establish context, and minimizing the time required to convey background information. The duration of each video is 4-5 minutes, and the content is designed to be concise and memorable for a diverse audience. The inclusion of a female doctor’s narration adds a layer of persuasiveness, reinforcing and promoting mental health awareness. One video is developed for each session to deliver the active therapeutic ingredients for fidelity and ensure delivery at the right dose.

This design approach not only ensures the accurate delivery of active elements through patient-computer interaction, but also maintains the crucial patient-facilitator interaction for counseling and empathy throughout the intervention process. In addition, TA-PM+ enhances the counseling skills of LHWs and the therapeutic alliance between LHWs and patients.

##### Web Dashboard for Supervisors

The online dashboard provides a user-friendly interface tailored for supervisors, streamlining the data from TA-PM+. This dashboard is structured to include an individual interface for each LHW, which in turn provides access to specific patient details. Supervisors can consistently monitor key information, such as session duration, delivery dates, and PSYCHLOPS scores. Moreover, the system incorporates notifications to alert supervisors in the event of a patient expressing suicidal thoughts, facilitating active case monitoring and prompt referral initiation.

#### Prototyping and Usability Testing

Employing the MUAQ designed for standalone mHealth apps used by health care providers, usability testing was performed twice, and it involved a cohort of 6 LHWs.

In the initial evaluation, 6 participating LHWs (mean age 44 years; range 41-58 years) were invited to fill the questionnaire and provide their feedback, which highlighted the issues of user interface, language, and pop-up alert settings.

The MUAQ item scores are shown in Table 2. After training, the mean usability score was recorded at 5.62 (highest possible score of 7). In the subsequent field assessment, the usability score increased to 5.96, which indicates improvement resulting from the incorporation of input from the first round. The usability scores demonstrated the system’s overall usefulness.

Table 2 displays the average overall scores of the MUAQ according to the following 3 categories: ease of use, interface and satisfaction, and usefulness for delivering TA-PM+. After incorporating feedback from round 1 of usability testing into TA-PM+, scores of the categories ease of use and interface satisfaction increased in round 2. However, scores of the category usefulness for delivering PM+ did not change. This may be because comments were largely sought on the usability, interface, and functionality of TA-PM+ and not on its content, which was adapted from the PM+ manual.

In the subsequent assessment phase, field usability testing was conducted with 6 participants who were screened for depression using the GHQ-12 questionnaire and WHODAS. The 6 screened participants (mean age 29 years; range 25-37 years) were invited to fill the mHealth Satisfaction Questionnaire after TA-PM+ session delivery by LHWs. The TA-PM+ session client satisfaction score was 40 (highest possible score of 46) (Table 3), indicating that the patients expressed contentment with the session.

**Table 2:** Usability scores of the mHealth Usability App Questionnaire (MUAQ) for the Technology-Assisted Problem Management Plus (TA-PM+) app.

MUAQ^a^ item			Score, mean (SD)			
			Round 1 (posttraining)		Round 2 (field)	
**Ease of use**			4.33 (0.31)		5.13 (0.47)	
	The app was easy to use.	4.50 (1.97)		4.33 (2.65)		
	It was easy for me to learn to use the app.	5.00 (1.78)		5.83 (1.83)		
	The navigation was consistent when moving between screens.	3.00 (2.28)		5.16 (2.56)		
	The interface of the app allowed me to use all the functions (such as looking at the dashboard, filling the PSYCHLOPS^b^, opening different sessions, and viewing videos) offered by the app.	4.16 (2.40)		4.00 (2.50)		
	Whenever I made a mistake using the app, I could recover easily and quickly.	5.00 (1.67)		6.33 (1.63)		
**Interface and satisfaction**			6.01 (0.67)		6.21 (0.51)	
	I like the interface of the app.	5.16 (2.10)		5.83 (2.85)		
	The information in the app was well organized, so I could easily find the information I needed.	5.33 (1.86)		6.33 (1.63)		
	The app adequately acknowledged and provided information to let me know the progress of my action (delivered sessions).	6.33 (1.63)		6.33 (1.54)		
	I feel comfortable using this app while delivering the session to a patient.	5.83 (1.83)		6.33 (1.21)		
	The amount of time involved in using this app has been fitting for me.	6.33 (1.63)		6.33 (1.63)		
	I would use this app again.	7.00 (0.00)		6.33 (1.63)		
	Overall, I am satisfied with this app.	6.33 (1.63)		6.33 (1.63)		
**Usefulness**			6.11 (0.89)		6.11 (0.61)	
	The app would be useful for my health care practice.	7.00 (0.00)		6.33 (1.63)		
	The app improved my access to delivering mental health service.	7.00 (0.00)		6.33 (1.63)		
	The app helped me manage my patients’ mental health effectively.	6.33 (1.21)		6.16 (1.60)		
	This app has all the functions and capabilities I expected it to have.	6.00 (1.26)		6.50 (1.22)		
	I could use the app even when the internet connection was poor.	3.66 (2.65)		4.50 (2.25)		
	This mobile health app provides an acceptable way to deliver the PM+^c^ intervention, such as accessing educational materials, tracking my own activities, and performing assessments.	6.66 (0.51)		6.83 (0.40)		
Overall usability of the TA-PM+^d^ app			5.62 (0.81)		5.96 (0.59)	

^a^MUAQ: mHealth Usability App Questionnaire.

^b^PSYCHLOPS: Psychological Outcome Profiles.

^c^PM+: Problem Management Plus.

^d^TA-PM+: Technology-Assisted Problem Management Plus.

**Table 3:** Client satisfaction (mHealth Satisfaction Questionnaire) with the videos and sessions delivered by lady health workers using the Technology-Assisted Problem Management Plus (TA-PM+) app.

mHealth Satisfaction Questionnaire items	Score, mean (SD)
The video was helpful in receiving the sessions.	4.83 (0.40)
I enjoyed receiving the sessions through the app and videos.	5.00 (0.00)
The time spent receiving the session through the app was acceptable.	4.83 (0.40)
The language used in the videos was difficult to understand.	5.00 (0.00)
The information given in the video was in small chunks, which I was able to retain.	4.83 (0.40)
Videos were too time consuming.	2.33 (2.06)
I didn’t like the videos or app usage during the session, and it felt like interruption.	1.66 (1.63)
It was so boring that I lost my interest during the sessions.	1.66 (0.81)
It was like a lecture, a disturbance, or too difficult to understand.	1.66 (0.00)
I can recommend it to others.	4.33 (1.03)
The avatars helped me to engage with the program. The scene settings were relatable, and it has motivated me to do the practices recommended for my well-being.	4.83 (1.02)
The examples in the videos helped me in understanding the information and helped me understand how the sessions will help my mental well-being.	4.83 (0.40)
The examples in the videos helped me understand how I need to implement session strategies in my life to feel better.	4.66 (0.51)
The videos and sessions have helped me set personal goals for my lifestyle habits in a way that I could not have done on my own.	4.83 (0.40)
Overall client satisfaction	40

## Discussion

### Principal Findings

The TA-PM+ app was designed to support LHWs in delivering the transdiagnostic PM+ intervention to patients with symptoms of stress, anxiety, and depression in Islamabad, Pakistan. The development process followed a thorough 3-stage methodology involving co-production with local stakeholders. Throughout the development of TA-PM+, careful consideration was given to the local health system perspective, available resources, and cultural sensitivities by involvement of experts. Usability testing confirmed that TA-PM+ is well-received by both LHWs and participants in community settings.

Urdu-translated PM+ was further adapted for technology-assisted delivery in our study using a comprehensive methodology involving local stakeholders and experts, a strategy recommended in the existing literature for PM+ adaptation. According to Heim et al [33], PM+ requires many adaptations to fit a given context before testing or implementing PM+ in that setting. Even changes to peripheral components of the intervention, such as instructional materials and illustrations, require the involvement of numerous experts in planning, translation, and cultural adaptation [34]. This procedural diligence is equally important when adapting the delivery style, for example, when PM+ is predominantly administered through web-based communication or when the length of treatment deviates from the originally specified 5 PM+ sessions [34].

The TA-PM+ app was developed through a co-production methodology, with the chosen delivery format being face-to-face interaction. The primary objective of the TA-PM+ app is to support LHWs in delivering the PM+ intervention in person, and patients only interact with the app to access psychoeducational videos. This particular design choice was carefully considered during the co-production to ensure a smooth integration with contextual elements and local digital and health resources, and the potential for a wider impact. Existing literature emphasizes that the success of digital technologies in mental health is dependent upon their alignment with sociopolitical conditions and cultural contexts. Failure to align with these factors often results in reduced user engagement and higher attrition rates. The term “nonalignment” encompasses various factors, such as values, mental health treatment methods, and specific health needs of the population [33,35].

The barriers to implementing TA-PM+ that were highlighted during the first round of qualitative investigation included skills and knowledge of LHWs to use TA-PM+, workload, time to implement TA-PM+, stigma associated with mental health, and lack of resources like internet availability. These barriers are in line with the current literature. A survey to explore barriers to implementation of digital mental health interventions in primary health care settings identified the availability of supporting infrastructure, organizational readiness and capacity, health care professional skills, and cultural appropriateness [36]. Research on digital mental health implementation [34] and digital health interventions through health systems [37] has shown that resource availability is a major barrier. The challenge posed by limited resource availability is particularly heightened in LMICs that are already struggling with resource constraints. A notable challenge in LMICs pertains to the availability of internet access and sufficient bandwidth. Addressing this barrier in digital health technologies is imperative, as highlighted both in our study and in a concurrent investigation conducted in Nepal [38,39], advocating for the implementation of interventions that are independent of internet connectivity. One of the most frequent recommendations for TA-PM+ that emerged through the qualitative investigation was to shorten the sessions from 90 minutes to 45-60 minutes to increase feasibility. This is consistent with the existing research on the reasons for high attrition in PM+. Prior research has indicated that participants commonly had difficulties finishing all 5 sessions of PM+ because of the lengthy sessions, which frequently interfered with finding or maintaining a job, performing domestic duties, or raising children [8-10]. Top of Form

During the co-production workshop, customization of the content, especially tailoring of the psychoeducational videos to respect the cultural nuances and demographics of the target communities, was planned for TA-PM+, to address stigma and improve engagement. Likewise, recent reviews on engagement with digital mental health interventions [40,41] identified that tailored digital technologies with personalized videos and avatars increased user engagement and demonstrated effectiveness. To augment persuasiveness in TA-PM+, there was a strategic decision during co-production workshops to have the videos narrated by a fictional doctor avatar. This design feature of TA-PM+ is consistent with the self-help digital intervention recently developed by the WHO for depression, wherein all the videos feature a doctor as the narrator [19].

### Limitations

There are limitations in this study, which have been divided into methodological limitations and design limitations of TA-PM+.

Methodological limitations include the potential lack of inclusivity and diversity in the co-production workshop sample, where people with lived experiences were not involved in the development of TA-PM+. Another limitation is the usability testing, which was conducted on a limited sample size and in the first session of TA-PM+, assuming interface uniformity across all sessions. The subsequent stage of our evaluation (feasibility study) will assess the feasibility, effectiveness, and usability of TA-PM+.

One of the main limitations in the design of TA-PM+ is that it requires the internet for the backend data storage function. Internet availability and bandwidth were identified as major barriers to the implementation of TA-PM+ in qualitative investigation. However, owing to backend storage of data and online monitoring, TA-PM+ cannot function without the internet, although some features requiring a high bandwidth, such as video playback, work without internet access. An additional limitation is that TA-PM+ will be administered by LHWs who provide services to women only. Thus, it will not be applicable to men, even though men require mental health care. TA-PM+ is currently in Urdu, necessitating technical and cultural adaptations to ensure its relevance and effectiveness in various sociocultural settings.

### Conclusions

We successfully developed the TA-PM+ app for LHWs, with the potential to be integrated into primary health care to address common mental health conditions. This work has several implications even beyond the Pakistan setting. These include (1) an understanding of the process of developing a resource-appropriate digital health tool for high uptake; (2) a model for adaptation that is flexible and can be used to create similar tools for PM+ in other low-resource settings globally; and (3) the ability of the system to be expanded to function as a national tool to capture the prevalence of common mental health conditions at the population level.

Further feasibility testing and eventual up-scaling are envisaged based on the thorough development process outlined in this paper.

## Appendix

Multimedia Appendix 1 COREQ (COnsolidated criteria for REporting Qualitative research) Checklist.

Multimedia Appendix 2 Interview guides.

Multimedia Appendix 3 Code chart.

## Abbreviations

COM-B: Capability, Opportunity, and Motivation for Behavioral Change
GHQ-12: General Health Questionnaire-12
LHS: lady health supervisor
LHW: lady health worker
LMIC: low- and middle-income country
mhGAP: Mental Health Gap Action Programme
MUAQ: mHealth App Usability Questionnaire
OPD: outpatient department
PM+: Problem Management Plus
PSQ: Patient Satisfaction Questionnaire
TA-PM+: Technology-Assisted Problem Management Plus
TDF: Theoretical Domain Framework
UC: Union Council
WHO: World Health Organization
WHODAS: WHO Disability Assessment Schedule

## Data Availability

All data generated and analyzed during this study are included in the main manuscript and its supplementary materials. The data are not deposited in a public repository due to the sensitive nature of participant information and ethical restrictions. Subject to ethical guidelines and institutional review board approval, the corresponding author can provide additional supporting data, such as comprehensive usability scores and satisfaction ratings, upon reasonable request.
