# Supplementary material for: A Digital Tool (Technology-Assisted Problem Management Plus) for Lay Health Workers to Address Common Mental Health Disorders: Co-production and Usability Study in Pakistan
Source: JMIR Form Res. 2025 Jan 28;9:e59414. doi: 10.2196/59414 (PMC11815293; doi:10.2196/59414)
Supplement: Multimedia Appendix 2 [file formative_v9i1e59414_app2.docx]

**Multimedia Appendix 2. Interview guides.**

**Aims**:

*TO GAIN INSIGHT INTO* ***C****APABILITIES,* ***O****PPORTUNITIES****, M****OTIVATIONS UNDER:*

1. LHWs capacity and capability for delivering a technology assisted PM+
2. Prior experience with government-issue “digital diaries”, and the technological needs and preferences of LHWs for developing an acceptable and feasible TA-PM+
3. Explore oppurtunities, barriers and facilitators to implement TA-PM+
4. the challenge of engaging male participants in the study

| **PROCESS**  *BEFORE THE INTERVIEW STARTS*   - Thank the participant for their time and contribution. (The duration of interview will be approx. 45 mins). - Say that we are interested in learning their views and experiences of digital diaries, stress and mental health problems in the community and the current PHC response, and the problem of delivering services to men in the community. - Check that the participant has read the Participant Information Sheet - Ask the participants if they have any questions about the interview and answer these. - Complete and sign consent form (2 copies, participant keeps one, researcher keeps one). - Reassure the participants that there are no right or wrong answers, we are really interested in their experiences and views so please be honest. Participation is anonymous and they will not be identified. - Tell them they can stop at any time. - Explain why we are using the recorder – because we can talk to each other directly, without the researcher having to write it done. Means we accurately record what you say, - Turn on recorder and start interview.   *AT THE END OF THE INTERVIEW*   - Thank participant again. - Inform them that if they wish to receive this, we will send them a summary of the study findings. |
| --- |

## Participant at Health system level

|  | **Preliminary Questions and General Characteristics of the Communities the LHWs serve:** |  |
| --- | --- | --- |
| 1.1 | What is scope of your work?  Prompts:  1. How long have you worked as an LHS? 2. How many LHWs under your supervision? 3. What is a typical workday like for you and for LHWs? What are their daily/weekly/monthly duties? How many households in the community do they cover? |  |
| 1.2. | What is your community like?  (Prompts: socioeconomic features: wealth, education, employment, household composition, cultural norms, social ties, neighborhood factors etc.) |  |
| 2. | **LHS/LHWs perceptions, training, and experience in mental health** | C |
| 2.1. | What kind of health problems do you most commonly encounter in the community?  Prompt:  1. Physical, psychological, social, etc.  2. Are there many in the community who seem stressed or express that they are mentally stressed?  3. How do people in your community usually manage stress or symptoms of “tension”? How is the problem viewed in the community?  4. How do LHWs handle that? Do they discuss this with you? | C |
| 2.2. | How does the primary healthcare system (LHWs, Primary Care Physician) respond to symptoms of stress and other psychological problems faced by people in the community?  Prompt:  1. Do people seek help or advise from you?  2. Which hospital/doctor people go to when they have stress related/mental health problems? | C |
| 2.3. | What do you understand by term “Mental health” and problems such as depression, anxiety?  Prompts  1. Do you and LHWS receive any training for this?  2. Can you recognize a person who is depressed or have anxiety?  3. Does your training include basic guidelines or information about referral procedure for patients with depression and anxiety | C |
| **3.** | **Experiences with Digital Diaries** | O |
| 3.1. | You have recently been provided with Android Tablets, what is the function of these tablets/which of your duties/records are now digitally updated?  1. How has the change affected your work? (e.g., is monitoring and supervision easier now or has not changed?  2. What is good about the digital diaries?  3. What is challenging about the digital diaries?  4. Any feature in current digital diaries that is very difficult and challenging for you or LHWs?  5. What could be done to improve your and LHWs’ experience with technology? (Any feature that could be added; easier, simpler interface; internet provision; etc.) | O |
| 4. | **TA-PM+** *We intend to create a digital job aid (TA-PM+) for LHWs to assist them in delivering a low-intensity mental health intervention for common mental health disorders. The TA-PM+ will be downloaded into the LHWs' tablets and will assist them in monitoring the patient's symptoms using automated tools. It will also assist LHWs in delivering the intervention's five sessions. These five sessions will be included in the TA-PM+, along with easy-to-use videos and infographics that will make it simple for LHWs to deliver the sessions. The TA-PM+ will be downloaded to your tablet as well and help you in supervising LHWs*  *For this study the patients will be identified by LHWs and screened by the team already. LHWs will be trained to use this TA-PM+ by the research team.* | M |
| 4.1. | What do you feel about this?  Prompts:  1. Will it be helpful or Beneficial for stressed people in the community?  2. Would you and LHWs want to participate in the implementation of TA-PM+?  3. What would encourage you and LHWs to be a part of a stress/mental health intervention delivery agent?  4. Will something like this make delivering the intervention easier for you and LHWs? | M |
| 4.2. | What suggestions do you have for this application?  Prompt:  1. What would be the most convenient features of that App? /What would the ideal application be like?  (ease of use, one-click, video-based, text-based, infographics/pictorial depiction, little dependence on LHWs own skill and knowledge, short duration vs long duration, few sessions vs many sessions, frequency, internet-independence, glitch-free, sharable content,)  2. Is there a way that LHWs can share videos with patients like WhatsApp etc? | M |
| 4.3. | What barrier do you think can occur while delivering this?  Prompt:  1. What problems you think you and LHWs can have while delivering an intervention with 5 sessions?  2. Any suggestion based on your current experience with digital diaries? | M |
|  | **Engaging men in the community** | O |
| 5.1. | Are LHWs in charge of any duties that include men in the households? | O |
| 5.2. | What do you think about delivering this intervention to men in community?  Prompt:  1. Do you think this is required in the community you work?  2. Who do you think is the best person to deliver such problem/stress management sessions to men? (you, LHWs, BHU doctor?)  2. What is your opinion about LHWs delivering stress-management sessions to men in the community? (Doable/feasible, appropriate according to their relationship with their community, appropriate according to sociocultural norms, etc.)  4. Is it possible for you to share some material via digital format to men of the community | O |

## Participant at Community level (LHWs)

| 1. | **Preliminary Questions and General Characteristics of the Communities the LHWs serve:** |  |
| --- | --- | --- |
| 1.1. | What is scope of your work  Prompt: 1 How long have you worked as an LHW? 2. What is a typical workday like? 3. What are your daily/weekly/monthly duties? 3. How many households in the community do you cover? |  |
| 1.2. | What is your community like?  (Prompts: socioeconomic features: wealth, education, employment, household composition, cultural norms, social ties, neighborhood factors etc.) |  |
| 2. | **LHWs perceptions, training, and experience in mental health** | C |
| 2.1. | What kind of health problems do you most commonly encounter in the community?  Prompt:  1. Physical, psychological, social, etc.  2. Are there many in the community who seem stressed or express that they are mentally stressed?  3. How do people in your community usually manage stress or symptoms of “tension”? How is the problem viewed in the community?  4. Do they discuss this with you? How do you handle that? | C |
| 2.2. | How does the primary healthcare system (LHWs, Primary Care Physician) respond to symptoms of stress and other psychological problems faced by people in the community?  Prompt:  1. Do people seek help or advise from you?  2. Which hospital/doctor people go to when they have stress related/mental health problems? | C |
| 2.3. | Does your training cover mental health problems, such as stress, anxiety, depression?  Prompt:  1. Do you understand what mental health problems are?  2. Can you recognize a person who is depressed or have anxiety?  3. Does your training include basic guidelines or information about referral procedure for patients with depression and anxiety | C |
| **3.** | **Experiences with Digital Diaries** | **O** |
| 3.1. | You have recently been provided with Android Tablets, what is the function of these tablets/which of your duties/records are now digitally updated?  Prompt:  1. How has the change affected your work?  2. What is good about the digital diaries  3. What is challenging about the digital diaries  4. Any feature in current digital diaries that is very difficult and challenging for you  5. What could be done to improve your experience with technology? (Any feature that could be added; easier, simpler interface; internet provision; etc.) | O |
| 4. | **TA-PM+ Description**  *We intend to create a digital job aid (TA-PM+) for LHWs to assist them in delivering a low-intensity mental health intervention for common mental health disorders. The TA-PM+ will be downloaded into the LHWs' tablets and will assist them in monitoring the patient's symptoms using automated tools. It will also assist LHWs in delivering the intervention's five sessions. These five sessions will be included in the TA-PM+, along with easy-to-use videos and infographics that will make it simple for LHWs to deliver the sessions.*  *For this study the patients for LHWs will be identified by the team already. LHWs will be trained to use this TA-PM+ by the research team.* | M |
| 4.1. | What do you feel about this?  Prompts:  1. Will it be helpful or beneficial for stressed people in the community?  2. Would you want to participate in the implementation of TA-PM+?  3. What would encourage you to be a part of a stress/mental health intervention delivery agent?  4. Will something like this make delivering the intervention easier for you? | M |
| 4.2. | What suggestions do you have for this application?  Prompt:  1. What would be the most convenient features of that App? /What would the ideal application be like?  (ease of use, one-click, video-based, text-based, infographics/pictorial depiction, little dependence on LHWs own skill and knowledge, short duration vs long duration, few sessions vs many sessions, frequency, internet-independence, glitch-free, sharable content,)  2. Is there a way to share videos with patients like WhatsApp etc.? | M |
| 4.3. | What barrier do you think can occur while delivering this?  Prompt:  1. What problems you think you can have while delivering an intervention with 5 sessions? | M |
| **5.** | **Engaging men in the community** | **O** |
| 5.1. | Are LHWs in charge of any duties that include men in the households? |  |
| 5.2. | What do you think about delivering this intervention to men in community?  Prompt:  1. Do you think this is required in the community you work?  2. Who do you think is the best person to deliver such problem/stress management sessions to men?  2. What is your opinion about LHWs delivering stress-management sessions to men in the community? (Doable/feasible, appropriate according to their relationship with their community, appropriate according to sociocultural norms, etc.)  4. Is it possible for you to share some material via digital format to men of the community | O |

## Participant at Health Policy level

| 1. | **Overview of existing systems in the context of developing and implementing TA-PM+** | **C** |
| --- | --- | --- |
|  | Scope of your work  Prompt:  1. What are your primary pursuits?  2. Is your work focused on noncommunicable diseases or mental health?  3. Is your work focused on health-care digitization?  4. Is your work focused on policy for LHWs?  One of question 1.2,1.3, 1.4 will be selected depending on scope of work. |  |
| 1.2. | What initiatives are being taken/ planned for mental health in Islamabad?  Prompts:  1. Are Mental health treatments now being integrated into health systems in a new way?  2. Anything new planned for coming years?  3. What about the UHC package how it targets mental health? | C |
| 1.3. | What initiatives are being taken/ planned for digitization in health in Islamabad?  Prompts:  1. Are digital initiatives now being integrated into health systems in a new way?  2. Anything new planned for coming years?  3. What about the UHC package how it targets digitization? | C |
| 1.4. | What new initiatives are being taken/ planned for lady health worker programs.  Prompts:  1. Are digital initiatives now being integrated in LHWs routine?  2. Any new inclusion of mental health services through them?  2. Anything related planned for coming year? | C |
| 1.5. | Can you tell some details about digital health strategy/ NCD strategy for the country/ UHC strategy? | C |
| 2. | **Suggestions, Potential Facilitators, and Barriers in Developing and Implementing TA-PM+**  We intend to create a digital job aid (TA-PM+) for LHWs to assist them in delivering a community-based low-intensity mental health intervention for common mental health disorders. The TA-PM+ will be downloaded into the LHWs' tablets and will assist them in monitoring the patient's symptoms using automated tools. It will also assist LHWs in delivering the intervention's five sessions. These 5 sessions will be included in the TA-PM+, along with easy-to-use videos and infographics that will make it simple for LHWs to deliver the sessions. |  |
| 2.1. | What are the opportunities for TA-PM+ to be integrated in LHWs schedule?  Prompt:  1. How do you think this can be successfully taken up by LHWs?  2. What are the current challenges faced by LHWs using digital diaries?  3. What type of challenges you think we can face while implementing?  4. What field issues we should consider while developing/ implementing this?  5. Are there any legislations/ strategy document that should be consulted by our team? | O |
| 2.2. | What skills will be required for LHWs to deliver this?  Prompts:  1. What type of training LHWs are given for using digital diaries?  2. What type of knowledge they have for mental health?  3. Apart from training for PM+ what other aspect training should cover? | C |
| 2.4. | How we can successfully implement this intervention  Prompt:  1. What will motivate LHW to deliver this service efficiently  2. Who are the key-players at policy level that should be contacted before implementing this?  3. What will motivate key-player (policy level) to integrate such service?  4. How this should be advocated for upscaling? | M |
|  | What type of features of TA-PM+ will help LHWs successfully delivering it?  Prompt:  1. Video-based, text-based, infographics/pictorial depiction, little dependence on LHWs own skill and knowledge, short duration vs long duration, few sessions vs many sessions, frequency, internet-independence, glitch-free, sharable content  2. Apart from features planned what type of features would complement the existing system of digital diaries?  3. Can LHWs share some material with the community digitally?  4. What features of digital diaries are LHWs finding difficult to use? | M |
| 4. | Engaging men in the community? | O |
| 4.1. | How do you think this service can be delivered to men in the community?  Prompt:  1. Do you think this is required in the community you work?  2. Who do you think is the best person to deliver such problem/stress management sessions to men?  3. What is your opinion about LHWs delivering stress-management sessions to men in the community? (Doable/feasible, appropriate according to their relationship with their community, appropriate according to sociocultural norms, etc.)  4. Is it possible for LHWs to share some material via digital format to men of the community? | O |
