# Supplementary material for: A Digital Tool (Technology-Assisted Problem Management Plus) for Lay Health Workers to Address Common Mental Health Disorders: Co-production and Usability Study in Pakistan
Source: JMIR Form Res. 2025 Jan 28;9:e59414. doi: 10.2196/59414 (PMC11815293; doi:10.2196/59414)
Supplement: Multimedia Appendix 3 [file formative_v9i1e59414_app3.docx]

**Multimedia Appendix 3.** Code chart.

| **COM-B^a^ model**  **component** | **Perceived Factors** | | | |
| --- | --- | --- | --- | --- |
| **TDF^b^**  **constructs** | **Barriers to implement TA-PM+** | **Description** | **App facilitators** | **Description** |
| 1. Physical Capability |  |  |  |  |
| - 1. Skills | Lack of Digital skills of LHWs | Skill in using a digital application and implementing mental health interventions for lady health workers, lady health supervisors, and other personnel at the PHC | User Guidance | Instructions on how to use TA-PM+ |
| - 1. Organizational commitment/ Time | High Workload of LHWs | Existing workload of lady health workers. | Time efficient App | The usage of TA-PM+ is not time consuming. |
| - 1. Skills development | Need of Training for LHWs | Training of lady health worker to provide skills to use the digital application and deliver the mental health intervention | Increase digital and mental health literacy | Increase the knowledge of TA-PM+ in LHWs |
| 1. Psychological Capability |  |  |  |  |
| - 1. Cognitive Capability | Cognitive capacity of provider | The cognitive skills, memory, and attention of the lady health worker to administer TA-PM+ | Easy to use | The TA-PM+ is easy to use and requires minimal cognitive input |
| - 1. Behavioral regulation |  |  | Promote enablement | The TA-PM+ fosters intended actions of LHWs, ensuring successful delivery of TA-PM+ |
| 1. Physical Opportunity |  |  |  |  |
| 3.1. Resources/ Infrastructure | Scarcity of Digital Infrastructure | Infrastructure required to implement digital mental health intervention like internet, tablets, computers, online server | Internet independent App | The TA-PM+ can operate without the use of internet |
| 3.2. Monitoring, supervision and support | Need for Supervision | A systematic and structured process aimed at overseeing and evaluating the performance, activities, and service delivery of lady health worker within a healthcare system. | Online Support | Possibility to contact supervisors through the TA-PM+ |
| 1. Social Opportunity |  |  |  |  |
| 4.1. Stigma |  |  | Persuasion | The App addresses the adverse perceptions and attitudes of patients related to mental health disorders and discourage them from seeking professional help. |
| - 1. Provider-patient relationship | Delivery to women of household | The relationship between a lady health worker and a patient essential for the effective delivery of an intervention and for fostering adherence to the provider's recommendations. | Promote patient engagement | Promote the intervention delivery time, interest and subjective experience of the patient. |
| 1. Automatic Motivation |  |  |  |  |
| - 1. Reinforcement | Incentives | Incentives in the form of feedback, rewards for the LHWs | Useful App | The TA-PM+ helps LHWs deliver the intervention efficiently |
| - 1. Advocacy | Sensitize the LHWs | Measures to encourage the implementation of mental health intervention by lady health workers within their communities. It involves raising awareness, providing education, and supporting access to mental health services. |  |  |
| 1. Reflective Motivation |  |  |  |  |
| - 1. Credibility | Repute in the community | The reputation and trustworthiness of lady health worker in the community as a result of service delivery. The maintenance of high standards for the quality and consistency of services provided by lady health workers to develop it. |  |  |

a: Capability, Opportunity, Motivation–Behaviour Model

b: Theoretical Domain Framework
